# β-Alanine Is an Unexploited Neurotransmitter in the Pathogenesis and Treatment of Alzheimer’s Disease

**DOI:** 10.3390/neurosci7010013

**Published:** 2026-01-15

**Authors:** Cindy M. Wozniczka, Donald F. Weaver

**Affiliations:** 1 Krembil Brain Institute, University Health Network, Toronto, ON M5T 1M8, Canada; 2 Department of Pharmaceutical Sciences, University of Toronto, Toronto, ON M5S 3H2, Canada; 3 Department of Medicine (Neurology), University of Toronto, Toronto, ON M5S 3H2, Canada; 4 Department of Chemistry, University of Toronto, Toronto, ON M5S 3H6, Canada

**Keywords:** Alzheimer’s disease, dementia, neurodegeneration, β-alanine, neurotransmitter, taurine, carnosine

## Abstract

Alzheimer’s disease (AD) remains an unmet medical challenge, as there are no effective therapies that alter the disease’s progression. While approaches have targeted molecules like acetylcholine (ACh) and glutamate, these strategies have provided only limited benefits and do not address the complex molecular mechanisms underlying AD development. This review suggests that β-alanine (3-aminopropanoic acid) is an underexplored neurotransmitter that could serve as a potential AD drug target. Existing evidence indicates that β-alanine modulates GABAergic and glutamatergic neurotransmission, thereby affecting neuronal hyperexcitability. Additionally, studies suggest that β-alanine has antioxidant effects, reducing oxidative stress caused by reactive oxygen species (ROS). We propose that β-alanine might bind to Aβ/tau proteins, possibly targeting the six-amino acid sequences EVHHQK/DDKKAK, which are involved in protein aggregation. β-Alanine may also influence the release of pro-inflammatory cytokines from microglia, potentially reducing neuroinflammation. We also hypothesize that β-alanine may help regulate metal dyshomeostasis, which leads to ROS production. Taurine, structurally like β-alanine, appears to influence comparable mechanisms. Although structural similarity doesn’t ensure therapeutic effectiveness, this evidence supports considering β-alanine as a treatment for AD. Furthermore, β-alanine and its analogues face challenges, including crossing the blood–brain barrier (BBB) and optimizing structure–activity relationships (SAR). This review includes articles through September 2025, sourced from four databases.

## 1. Introduction

Dementia impacts over 50 million people worldwide and is expected to triple by 2050, with Alzheimer’s disease (AD) being the most common type [1,2,3]. Despite significant efforts to find an effective treatment for AD, there is still no long-term cure available [4,5]. Current therapeutics (Table 1) for AD are not curative. These non-curative treatments fall into two main categories: symptomatic relief and disease-modifying therapies that aim to slow down disease progression. Symptomatic treatments include N-methyl-D-aspartate (NMDA) receptor antagonists, such as memantine, and cholinesterase inhibitors, such as donepezil, galantamine, and rivastigmine [6,7]. Current potentially disease-modifying therapies include biologics such as Lecanemab and Donanemab [8,9,10]. These two biologics can reduce Aβ plaque formation but have significant side effects [8,9,10]. Current therapeutic approaches for AD do not offer a cure, highlighting the need for new therapeutic targets.

This review examines how β-alanine functions as a neurotransmitter and its potential to target different mechanistic sites involved in AD pathogenesis, offering a new avenue for AD research. However, this review is speculative and does not present a confirmed treatment strategy.

**Figure 1:**
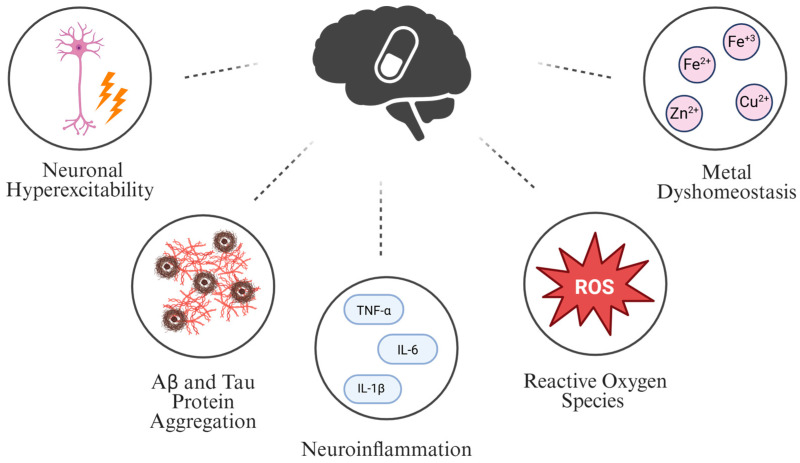
β-Alanine is believed to target multiple mechanisms involved in AD pathogenesis, including neuronal hyperexcitability, Aβ and tau protein aggregation, neuroinflammation, reactive oxygen species (ROS), and metal imbalance. These target sites should be addressed to develop an AD treatment Created in BioRender. Wozniczka, C. (2026) https://BioRender.com/fvjqvhx.

A safe and curative treatment for AD remains an unachieved goal, emphasizing the need for new therapeutic approaches. This review suggests that β-alanine (3-aminopropanoic acid) is a promising target for AD drug research. β-Alanine and its receptors are possible druggable targets for AD because they interact (directly and indirectly) with multiple disease-relevant mechanistic sites. These mechanistic sites include neuronal hyperexcitability, Aβ and/or tau protein misfolding/oligomerization, neuroinflammation, reactive oxygen species (ROS) production, and metal dyshomeostasis (Figure 1). Additionally, β-alanine is suggested to be a good target for AD because it acts as a structural intermediate between glycine and GABA—two inhibitory neurotransmitters in the central nervous system (CNS) [11]. Various neurotransmitters like acetylcholine (Ach), GABA, and glutamate have been investigated as AD therapies; none have proven successful. Thus, β-alanine has the potential to be the next neurotransmitter under investigation for the treatment of AD. β-Alanine is also a precursor to carnosine, a molecule with neuroprotective properties [11], and it can cross the blood–brain barrier (BBB) via active carrier-mediated transport [12,13,14,15,16,17,18]. β-Alanine thus offers several benefits as a drug target for AD research.

Although β-alanine may be considered a promising research target, it also has significant limitations that warrant consideration. For instance, most studies reported in the literature are based exclusively on in vitro and in vivo data. The few available human studies tend to be small, focused on β-alanine as a nutritional supplement and often restricted to body-builders or endurance athletes. It is also important to note that most of these studies were conducted in specific geographical regions, and did not encompass all demographic groups. Also, AD is also a multifactorial disease; therefore, targeting the multiple mechanistic sites involved in AD pathogenesis poses significant challenges.

## 2. Methodology

### 2.1. Literature Search Strategy

For this review, a thorough literature search was conducted using the electronic databases PubMed, Web of Science, Scopus, and Google Scholar, covering articles published up to September 2025. Electronic databases were searched with combinations of keywords related to β-alanine and Alzheimer’s disease, using Boolean operators (AND/OR). Articles were chosen based on their relevance to the topic and scientific significance. The keyword included (“beta-alanine” OR “β-alanine”) AND (“neurotransmitter OR synaptic OR “synaptic transmission” OR GABA OR glutamate OR acetylcholine”), (“beta-alanine” OR “β-alanine”) AND (“Alzheimer’s disease” OR Alzheimer) AND (“neuronal hyperexcitability” OR excitability OR “neuronal firing” OR “action potential” OR epilepsy OR seizure* OR “ion channel”), (“beta-alanine” OR “β-alanine”) AND (“Alzheimer’s disease” OR Alzheimer) AND (“amyloid beta” OR “Aβ” OR amyloid) AND (aggregation OR oligomer OR fibril OR plaque), (“beta-alanine” OR “β-alanine”) AND (“Alzheimer’s disease” OR Alzheimer) AND (tau OR “tau protein”) AND (aggregation OR hyperphosphorylation OR fibril OR tangle), (“beta-alanine” OR “β-alanine”) AND (“Alzheimer’s disease” OR Alzheimer) AND (neuroinflammation OR inflammation OR microglia OR astrocyte OR cytokine OR “TNF-alpha” OR interleukin), and (“beta-alanine” OR “β-alanine”) AND (“Alzheimer’s disease” OR Alzheimer) AND (“reactive oxygen species” OR ROS OR oxidative OR “oxidative stress” OR antioxidant), (“beta-alanine” OR “β-alanine”) AND (“Alzheimer’s disease” OR Alzheimer) AND (“metal dyshomeostasis” OR “metal imbalance” OR iron OR copper OR zinc OR “metal chelation”).

This article was prepared as a narrative literature review. While relevant studies were found through database searches, the exact numbers of records excluded, included, and identified were not systematically recorded because the PRISMA-guided selection process was not employed.

### 2.2. Inclusion and Exclusion Criteria

Eligible studies encompassed in vitro and in vivo research, such as neuronal/glial cell models and animal models displaying relevant AD pathology. Human studies were considered if β-alanine was supplemented to individuals with CNS-related diseases, mild cognitive impairment (MCI), or AD. The review included investigations of β-alanine’s connection with receptor sites, neuronal hyperexcitability, Aβ and tau aggregation, neuroinflammation, reactive oxygen species (ROS) production, and metal dyshomeostasis. Studies focusing on β-alanine’s biochemistry, metabolism, transport, and related molecules like carnosine and taurine were also included. Since this is a comprehensive review, we have rigorously included studies from the 1950s to the 1980s that report some of the earliest work on β-alanine AD receptor sites. These studies, though dated, remain seminal in this area. Furthermore, only articles in English with full text and clear relevance to β-alanine as a neurotransmitter, AD, and its therapeutic potential were considered.

Studies were excluded only if they focused on outcomes unrelated to the CNS. Letters, case reports, and articles without full text or not in English were also excluded.

### 2.3. Data Extraction

The study selection process followed a defined screening protocol. All titles and abstracts were reviewed for their relevance to β-alanine’s biochemistry, its role as a neurotransmitter, its relevance to AD, and its connection to the five mechanistic sites mentioned earlier. Full-text articles were subsequently evaluated and selected based on specific inclusion and exclusion criteria. Data extraction was conducted using standardized methods to maintain consistency across all studies.

### 2.4. Quality of Assessment

This narrative review evaluated the quality of evidence using a flexible, structured approach that suits its descriptive scope. Sources were assessed for publication credibility (such as peer-reviewed journals), clarity of study design, transparency of methods, and relevance to the review topic. Preference was given to well-structured studies with clear methods, adequate sample sizes, and consistent results across multiple sources. Limitations like bias, small sample sizes, or incomplete data were noted and considered during interpretation. Instead of a formal scoring system, evidence quality was compared across studies to enable a balanced synthesis, recognizing differences in methodological rigor.

## 3. Biochemistry of β-Alanine

### 3.1. Biosynthesis of β-Alanine

**Scheme 1:**
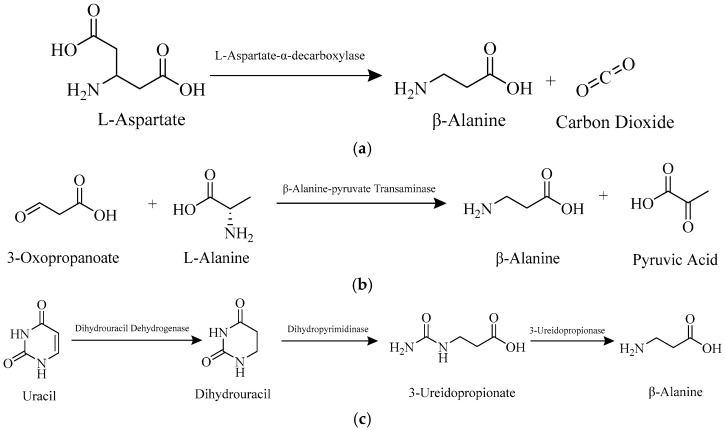
Three metabolic pathways for the biosynthesis of β-alanine. (**a**) Decarboxylation of L-aspartate by gut microbes to form β-alanine [11,20,21,22,23]. (**b**) 3-Oxopropanoate and L-alanine are converted to β-alanine and pyruvic acid [11,20,24,25]. (**c**) Uracil is converted into dihydrouracil, which is then converted into 3-ureidopropionate and β-alanine [11,20,26,27]. Impaired β-alanine biosynthesis could contribute to AD.

In humans, the primary dietary source of β-alanine is beef or fish [19]. If not consumed through food, β-alanine is biosynthesized via three metabolic pathways [11,20]. The first involves the decarboxylation of L-aspartate by gut microbes [11,20,21,22,23]. Bacteria in the microbiome have L-aspartate-α-decarboxylase (PanD), which converts L-aspartate into β-alanine and carbon dioxide (Scheme 1) [11,20,21,22,23]. Another pathway for β-alanine production involves a reversible reaction between 3-oxopropanoate (malonic semialdehyde) and L-alanine, resulting in pyruvic acid and β-alanine [11,20,24]. This reaction is catalyzed by β-alanine-pyruvate transaminase (β-alanine-α-alanine transaminase) in the liver (Scheme 1) [11,20,24,25]. The final pathway involves the breakdown of pyrimidine nucleotides (Scheme 1), especially uracil, in the liver [11,20,25,26,27]. Uracil is converted to dihydrouracil, then to 3-ureidopropionate, and finally to β-alanine [11,20,26,27]. These steps are catalyzed by dihydrouracil dehydrogenase (dihydropyrimidine dehydrogenase), dihydropyrimidinase (dihydropyrimidine amidohydrolase), and 3-ureidopropionase (β-alanine synthase), respectively [11,20,26,27]. Some sources suggest an alternative pathway in which β-alanine is formed from the breakdown of carnosine into β-alanine and L-histidine in the liver, catalyzed by carnosinase [28,29]. There are, therefore, multiple pathways in the body for producing β-alanine [11,20,21,22,23,24,25,26,27,28,29].

### 3.2. Transport of β-Alanine into Skeletal Muscle and Cardiac Tissue

β-Alanine is quickly absorbed into the bloodstream after ingestion or production in the gastrointestinal (GI) tract or liver [19,30,31]. β-Alanine moves from the GI tract into the bloodstream via enterocytes on the apical side of the small intestine [19,31]. It uses a proton-assisted amino acid transporter (PAT1) or taurine transporter (TauT) to enter these cells [19,31]. Inside the enterocyte, β-alanine is transported into the blood via a β-amino acid-specific transporter (βAAT) [19,31]. The pH level in the GI tract influences the absorption of β-alanine [32]. When the GI pH is 6.0, more β-alanine is absorbed, highlighting the role of the proton transporter [32]. Similarly, just as β-alanine leaves the GI tract to enter the bloodstream, the same process occurs in the liver, which removes simple amino acids like β-alanine using amino acid transporters [30]. Any β-alanine not absorbed into the bloodstream is directed to the kidneys [33,34,35]. In the kidneys, the renal brush-border membrane on proximal tubule cells contains sodium- and chloride-dependent transporters that regulate the amount of β-alanine reabsorbed into the bloodstream and excreted into the urine [33,34,35]. Once β-alanine is available in the bloodstream, it can circulate to its target organs [29,36]. Specifically, β-alanine enters skeletal muscles by crossing the sarcolemma through the TauT transporter [36]. This transporter is a sodium- and chloride-dependent channel that requires two sodium ions and one chloride ion to move β-alanine into the sarcolemma [36]. β-Alanine is also transported into skeletal muscle via the PAT1 transporter. However, TauT remains the primary transporter for transferring β-alanine into muscle tissue [36]. Similarly, β-alanine enters cardiac tissues through cardiomyocytes via TauT transporters [29]. Once β-alanine enters skeletal muscle and/or cardiac tissue, de novo synthesis occurs to form carnosine [20,28,29,37]. Numerous macromolecular components are involved in transporting β-alanine from its source, the GI tract and the liver, to non-neural target organs where it performs its primary functions [32,33,34,35,36].

### 3.3. Transport of β-Alanine into the Brain

For β-alanine to be present in the CNS, it must cross the BBB. β-Alanine crosses the BBB through active or carrier-mediated transport via the so-called “beta-shuttle” [12,13,14,15,16,17,18]. Komura et al. examined how β-amino acids are transported into the brain using bovine brain capillary endothelial cells [12,13,17,18]. This suggests that β-alanine uses carrier-mediated transport to cross the BBB [12,13,17,18]. Additionally, studies have been conducted to understand the ions and stoichiometric ratios needed for β-alanine carrier-mediated transport [17,38,39]. Rat brain membrane vesicles were utilized to create an artificial ion gradient to study β-alanine uptake [38]. This research demonstrated that β-alanine transport depends on sodium and chloride ions [38]. Other studies indicate that two sodium ions and one chloride ion are required to transport one β-alanine molecule across the BBB [18,39]. This aligns with another study on β-alanine transport into embryonic chick pectoral muscle [40]. It is clear that β-alanine crosses the BBB through active transport mechanisms involving sodium- and chloride-dependent ion channels [12,13,14,15,16,17,18,38,39,40].

### 3.4. Biodegradation of β-Alanine

The body can excrete β-alanine through urine or convert it into carnosine [41,42,43]. Harris et al. administered β-alanine to six healthy male adults at doses of 10, 20, or 40 mg/kg of body weight [41]. When urine was analyzed, less than 5% of the administered β-alanine was detected. Additional pharmacodynamic assessments involved giving 3.2 or 6.4 g (dosed at 400 or 800 mg) of β-alanine for four weeks. The results showed a 40% increase in muscle carnosine levels. This indicates that some of the β-alanine is converted into carnosine, and any remaining β-alanine is excreted unchanged in the urine [41]. Stegen et al. conducted a similar study in seven healthy male adults for five weeks with a daily dose of 4.8 g [42]. The results demonstrated that less than 2% of β-alanine was detected in urine, and only 3–6% was converted into carnosine. The same authors indicate that most of the administered β-alanine was metabolized via oxidation [42]. Other research by Pihl and Fritzson found that 90% of ^14^C-labelled β-alanine in rats was converted into carbon dioxide [43]. They concluded that β-alanine must be converted and utilized in other regions of the rat [43]. Only a small fraction of β-alanine is removed unchanged or converted into carnosine, whilst the remainder serves alternative functions in the body [41,42,43].

It is clear that β-alanine is not only excreted in the urine or converted into carnosine but also serves other functions in the body [43]. One proposed mechanism for this conversion involves the transamination of the amine group on β-alanine within the citric acid cycle to produce keto-acid malonate semi-aldehyde (MSA) [44,45,46]. This reaction is catalyzed by two mitochondrial enzymes: 4-aminobutyrate-2-oxoglutarate transaminase (also known as GABA-T or β-alanine-2-oxoglutarate transaminase) and alanine-glyoxylate transaminase (also called AGXT2 or β-alanine-pyruvate transaminase) [44,45,46]. The product, malonate semi-aldehyde, is CoA-dependent and undergoes further reactions to form acetyl-CoA [47]. Acetyl-CoA plays an essential role in metabolism and energy production in the citric acid cycle [48]. Other studies, such as those by Wilson et al., showed that when β-alanine was supplemented to rats, levels of 2-(aminomethyl)-malonate (AMM) increased in the liver [49]. These researchers suggested that when β-alanine is carboxylated by pyruvate carboxylase, using either acetyl-CoA or propionyl-CoA as activators, AMM is produced as a byproduct. AMM, a derivative of aspartate, is involved in neurotransmission. It was concluded that AMM produced from β-alanine intake might participate in neuronal metabolism [49]. This implies that β-alanine is converted into various metabolites, possibly for energy generation and neuronal function [44,45,46,47,48,49].

Another way β-alanine is degraded is through its reuptake by neurons and/or glial cells [50,51,52,53,54]. Robitaille et al. subjected male Sprague-Dawley rats to a sub-pial iontophoresis of iron(III) chloride (FeCl_3_) into the right motor strip to induce reactive glial cells [50]. These rats and the control group were supplemented with β-[14C]alanine to assess if glial cells would absorb it. The results showed that β-[14C]alanine was taken up by both normal and reactive glia. The uptake of β-[14C]alanine was also increased when endogenous GABA was reduced in cortical tissue [50]. Schon and Kelly studied [3H]β-alanine uptake in sensory ganglia and the cerebral cortex [51]. Their study showed that [3H]β-alanine is found only in glial cells within sensory ganglia and the cerebral cortex. They also observed that the uptake of [3H]β-alanine in sensory ganglia is sodium-dependent [51]. Iversen and Johnston tested fifty-two different molecules to see if they could inhibit [3H]GABA uptake in rat cerebral cortex slices [52]. β-Alanine was found to inhibit [3H]GABA uptake in these slices. This suggests that β-alanine can be absorbed by rat cerebral cortex slices, playing a role similar to GABA [52]. Roseth and Fonnum conducted a study in which synaptic vesicles were collected from the brains of rainbow trout and chickens, and the uptake of different neurochemicals was measured [53]. Glutamate, GABA, glycine, and β-alanine were absorbed into vesicles in both species. Additionally, the amount of β-alanine uptake in these species was similar to that in rats. The uptake ratios resembled those in rat synaptic vesicles [53]. A study by Robitaille and Sherwin examined the uptake and response of [3H]β-alanine in reactive astrocytes with a scar of epileptogenic foci in rat motor cortex [54]. The results revealed an increase in grain count in the scar tissue treated with [3H]β-alanine. The authors suggest that the higher uptake of [3H]β-alanine in reactive astrocytes may be due to a decrease in GABAergic neurons in the scar tissue. From this, it can be concluded that β-alanine and GABA share the same carrier system in glial membranes, and amino acid transporters may become stimulated in reactive glial cells. They also reported that neutral amino acids, such as β-alanine, are transported from cerebral endothelial cells to glial cells [54]. There is evidence that reuptake of β-alanine occurs in neurons and glial cells, which may be a form of biodegradation [50,51,52,53,54].

In summary, β-alanine is eliminated through urinary excretion, converted into carnosine, catabolized by enzymes to produce compounds used in metabolism and energy production, and reuptake by neurons and glial cells [41,42,43,44,45,46,47,48,49,50,51,52,53,54].

### 3.5. Carnosine and Its Connection to β-Alanine

Carnosine is an endogenous dipeptide composed of β-alanine and L-histidine [19,28,29,31,55,56]. Its synthesis is catalyzed by adenosine triphosphate (ATP)-dependent carnosine synthetase [31]. The production of carnosine depends on the level of β-alanine in the body [20,28,29,37,44]. Athletes often supplement with β-alanine to enhance the natural synthesis of carnosine in skeletal muscles, which is speculated to help reduce fatigue [37,57]. Injecting β-alanine has been suggested as a procedure to delay the onset of neuromuscular fatigue [37]. Carnosine is primarily found in skeletal muscles and heart tissue [31,56]. In skeletal muscles, it acts as a physiological buffer during high levels of lactic acid arising from exercise and exertion [28,29]. The imidazole ring on carnosine has a pKa of 6.83, allowing it to accept protons and reduce muscle acidity [28,29]. Additionally, in skeletal muscle and cardiac tissue, carnosine exhibits antioxidant properties; the imidazole ring can serve as an electron donor to help prevent lipid peroxidation [28,29,58]. It also chelates copper and iron, preventing ROS formation, regulating enzymes, and controlling calcium levels in the sarcoplasmic reticulum and myocardium [28,29,58]. Carnosine serves mainly roles in skeletal and cardiac tissues [28,29,58].

In addition to carnosine’s roles in skeletal muscle and cardiac tissue, carnosine also functions in the brain [28,29,58]. It may act as an endogenous neuromodulator or antioxidant, and it is suggested that it could serve as a neuroprotective agent for the CNS [59]. Carnosine can cross the BBB and is also synthesized within the brain [55,59]. Any excess carnosine not utilized by the body is broken down by carnosinase [55]. There are two isoforms of this enzyme: serum carnosinase (CN1) and tissue carnosinase (CN2). CN1 is present in both serum and the brain and degrades carnosine quickly. CN2 is manganese-dependent and is called a “cytosol non-specific dipeptidase” [55]. Carnosine performs many functions in the human body and relies on β-alanine for its biosynthesis [19,20,28,29,31,37,55,56,57,59].

Ultimately, extensive knowledge has been accumulated about the biosynthesis [11,20,21,22,23,24,26,27,28,29] and the biodegradation of β-alanine [41,42,43,44,45,46,48,49,50,51,52,53,54]. A wealth of information also explains how β-alanine is transported in skeletal muscle, cardiac tissue, and the brain [12,13,14,15,16,17,18,32,33,34,35,39,40]. Furthermore, there is evidence that clarifies β-alanine’s role in carnosine biosynthesis and how carnosine functions in mammalian tissues [19,20,28,29,31,37,55,56,57,59].

## 4. β-Alanine Receptor Sites

### 4.1. Glycine Co-Agonist Site on the NMDA Complex (Strychnine-Insensitive)

NMDA receptors are cation channels that promote excitatory neurotransmission in the CNS [60,61]. NMDA receptors have a glycine site that is insensitive to strychnine [62]. Sarcosine, a pharmacologically active compound, used as an adjunct therapy for schizophrenia, therapeutically binds to the glycine co-agonist site, thereby modulating glutamate function [61]. Multiple studies suggest that amino acids, including β-alanine, can bind to this site on the NMDA receptor as well [60,62,63]. For example, Pullan and Powel examined hippocampal and cortical synaptic membranes for their ability to displace [3H]glycine [60]. β-Alanine was found to interact with and displace [3H]glycine at the strychnine-insensitive glycine site on the NMDA receptor [60]. Ogita et al. experimented to evaluate [3H]glycine binding to strychnine-insensitive glycine receptors in rat brain synaptic membranes [63]. The binding of [3H]glycine was displaced by many different amino acids, including β-alanine [63]. This suggests that β-alanine can bind to this receptor site [60,63]. Additionally, McDonald et al. studied the distribution of the glycine strychnine-insensitive site in rat brains using [3H]glycine [62]. Their results showed that [3H]glycine binding varied across brain regions, with the highest levels in the hippocampus, followed by the cerebral cortex, caudate putamen, thalamus, cerebellum, and brainstem. They also analyzed how displacers competed for binding; these displacers were stereoselective, with the following order: glycine > D-serine > D-alanine > L-serine > L-alanine. Although β-alanine was not tested in this study, L-alanine and D-alanine were examined, and both isomers were able to displace [3H]glycine [62]. It is evident that many amino acids, including β-alanine, can bind to the glycine strychnine-insensitive co-agonist site on the NMDA receptor [60,62,63].

### 4.2. Glycine Receptor Site (Strychnine Sensitive)

Glycine receptors are ligand-gated chloride channels that mediate inhibitory neurotransmission [64,65]. β-Alanine binds to the glycine site of the glycine receptor [60,66,67,68,69,70,71,72,73,74,75]. Pullan and Powel examined the displacement of [3H]strychnine on the glycine channel in spinal cord and brainstem synaptic membranes [60]. Glycine and β-alanine can displace and interact with the glycine receptor site [60]. Beyer et al. used strychnine to induce seizures in a rat model and administered various amino acids to ascertain if seizures would improve or stop [66]. β-Alanine displaced strychnine and reduced the number and duration of convulsions [66]. Schmieden and Betz showed that wild-type alpha 1 glycine receptors on Xenopus oocytes responded to β-alanine [67]. Tremblay et al. analyzed β-alanine, taurine, and glycine in neurons of the cat cortex to examine if they affected neuronal excitability [68]. β-Alanine and taurine decreased neuronal excitability, demonstrating that these are inhibitory molecules in the CNS [68]. Choquet and Korn, and Boehm et al. used whole-cell patch clamp techniques on cultured chick spinal neurons and embryonic chick paravertebral sympathetic ganglia, respectively, to evaluate if β-alanine activated the glycine receptor [69,70]. β-Alanine was found to interact with and activate the glycine receptor [69,70]. Krishtal et al. collected rat neurons from the medulla oblongata and hippocampus [71]. They found that responses were blocked by strychnine, but glycine, β-alanine, and taurine stimulated the neurons [71]. Furthermore, DeFeudis et al., Wu et al., Mori et al., and Laube et al. conducted studies demonstrating that β-alanine binds to glycine receptors in the brain [72,73,74,75]. Multiple data thus confirm that β-alanine binds to the glycine site on the glycine receptor [60,66,67,68,69,70,71,72,73,74,75].

### 4.3. GABA-A and GABA-C Receptors

GABA is a well-studied neurotransmitter [76]. There are three subtypes of GABA receptors: GABA-A, GABA-B, and GABA-C [76]. GABA-A and GABA-C receptors are ligand-gated chloride channels involved in inhibitory neurotransmission in the CNS [76,77]. A large body of evidence suggests that β-alanine binds to GABA-A receptors. 69,73,78,79,80]. For example, Wu et al. showed that β-alanine is comparable to GABA in its ability to bind GABA-A receptors, as demonstrated through electrophysiology [73]. Shin et al. examined different agonists binding to the GABA-A receptor [78]. β-Alanine produced a similar number of open channels as GABA, demonstrating that it can act as an agonist at the GABA-A receptor [78]. Jones et al. suggest that β-alanine is a low-affinity, high-efficacy agonist at the GABA-A receptor [79]. Choquet and Korn indicate that β-alanine acts as a partial agonist at the GABA-A receptor [69]. Martinez-Torres and Miledi used complementary deoxyribonucleic acid (cDNA) encoding the γ2L subunit of the human GABA-A receptor, which was injected into Xenopus oocytes; this receptor was found to be stimulated by β-alanine [80]. β-Alanine can interact with the GABA-A receptor, causing an inhibitory response in the CNS [69,73,78,79,80].

Studies also indicate that β-alanine interacts with GABA-C receptors similarly to GABA-A receptors [81,82]. Chesnoy-Marchais found that GABA-C receptors were activated by taurine and β-alanine in hypoglossal motoneurons [81]. Calvo and Miledi expressed GABA-C receptors in Xenopus oocytes and measured the conductance of this receptor [82]. These receptors were found to respond to glycine and β-alanine [82]. GABA-C receptors can bind β-alanine, leading to inhibitory neurotransmission [81,82].

Likewise, studies have also shown β-alanine’s ability to bind to GABA receptors without identifying the specific receptor subtype [83,84,85,86]. Horikoshi et al. studied what causes Xenopus oocyte injected with mouse brain messenger ribonucleic acid (mRNA) to respond when given taurine and β-alanine [83]. The response observed in brain mRNA from these molecules involved both GABA and glycine receptors [83]. Orensanz and Barrio prepared mRNA from Xenopus oocytes and found that β-alanine has a similar response to GABA at GABA receptors [84]. Brown and Scholfield tested the effect of GABA and agonists, including β-alanine, on single neurons in slices of guinea pig olfactory cortex using microelectrodes [85]. The membrane conductance was decreased by β-alanine, depolarizing the membrane but to a lesser extent than GABA [85]. Brown and Galvan examined potential changes in the membrane of guinea-pig olfactory cortex slices [86]. β-Alanine, among other agonists, was found to exhibit a decrease in electrical potential compared to GABA [86]. β-Alanine binds to GABA-A and GABA-C receptors, triggering inhibitory signals in the brain [69,73,78,79,80,81,82,83,85,86].

### 4.4. GABA Transporter (GAT) Protein-Mediated Glial GABA Uptake

Another receptor site for β-alanine in the CNS involves GAT proteins in GABA uptake systems. GATs are sodium- and chloride-dependent transmembrane proteins found on neurons and glial cells [87,88]. These transporters facilitate the movement of molecules throughout the CNS [11]. Several isoforms of GAT exist, including GAT-1, GAT-2, GAT-3, and GAT-4 [89,90]. GAT-1 mRNA is present throughout the brain, especially in the olfactory bulb, basal ganglia, interpeduncular nucleus, cerebellum, and retina [90]. GAT-1 proteins also occur in non-GABAergic and glial cells in some brain regions. GAT-2 mRNA shows low expression and is primarily found in brain arachnoid and ependymal cells. GAT-3 mRNA is observed in the glomerular layer of the olfactory bulb, the inner retina, the thalamic paraventricular nucleus, and the globus pallidus [90]. GAT-4 proteins are located in the olfactory bulb, cerebral cortex, thalamus, hippocampus, superior colliculus, inferior colliculus, substantia nigra, cerebellum, and brainstem [89]. Previous work by Eckstein-Ludwig, Fei, and Schwarz has shown that GABA uptake at pharmacological concentrations produces therapeutic effects for various antiepileptic drugs [88]. Likewise, GAT-3 and GAT-4 are known to bind and transport β-alanine at high concentrations in the CNS [11,91,92]. GAT-2 also binds and transports β-alanine throughout the CNS [11,92]. Furthermore, since GAT-2, GAT-3, and GAT-4 facilitate β-alanine transport, the level of β-alanine directly affects the transport of other molecules within the CNS [11,92,93]. Schon and Kelly demonstrated that β-alanine exhibits a similar affinity to GABA in glial cell uptake systems, but this affinity is less pronounced at nerve terminals [51]. [3H]β-Alanine was supplied to glial cells in sensory ganglia and slices of the cerebral cortex. [3H]β-Alanine was observed to accumulate and can be taken up by glial cells in sensory ganglia and slices of the cerebral cortex. Additionally, the competitive inhibition kinetics of GABA and [3H]β-alanine in cortical slices indicate that glial cells remain functional, allowing GABA to bind and be taken up. GAT proteins play a significant role by acting as a receptor to transport β-alanine across the CNS [51].

**Figure 2:**
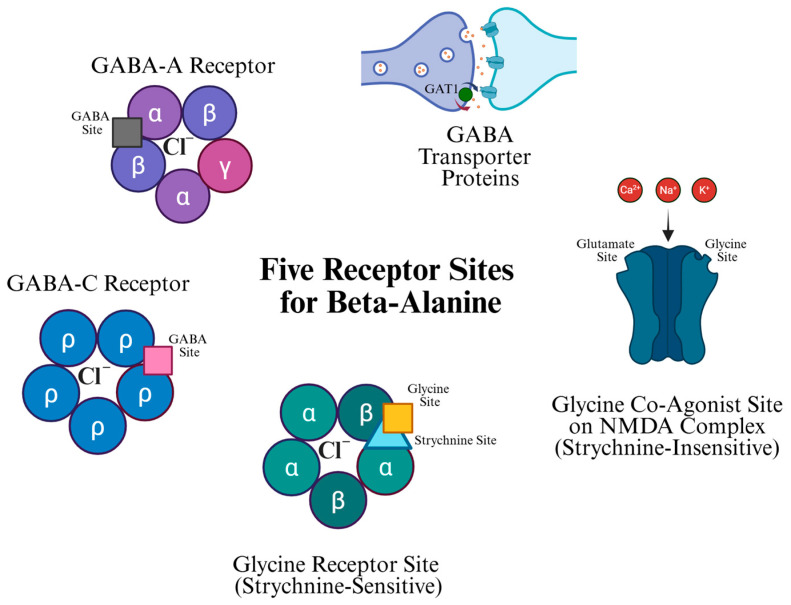
β-alanine has five known receptor sites in the body that may contribute to AD, including the glycine co-agonist site on the N-methyl-D-aspartate (NMDA) complex (strychnine-insensitive), the glycine receptor site (strychnine-sensitive), the GABA-A receptor, the GABA-C receptor, and the GABA transporter (GAT) proteins [11] Created in BioRender. Wozniczka, C. (2026) https://BioRender.com/1vuuy8m.

β-Alanine interacts with various receptors in the CNS (Figure 2), specifically NMDA, glycine, GABA-A, GABA-C receptors, and GAT proteins [11]. These receptors present significant potential for β-alanine to modulate both excitatory and inhibitory neurotransmission. This also emphasizes β-alanine’s effect on the CNS and its potential as a drug design platform.

## 5. β-Alanine as a Neurotransmitter

Neurotransmitters are crucial components in chemical synaptic transmission. Werman established criteria in 1966 to determine whether an endogenous chemical functions as a neurotransmitter [94]. Many well-studied neurotransmitters have been identified using these criteria [94]. Examples include Ach, GABA, glycine, and glutamate [11,95,96,97]. However, Werman’s criteria have become less significant over time, as nitric oxide has been classified as a neurotransmitter despite not meeting all these criteria [11]. Werman’s criteria for a molecule being a neurotransmitter include:

(1) Existence of inactivating enzyme(s);
(2) Existence of the transmitter in neural tissues;
(3) Ability for storage of the transmitter in neural tissues;
(4) Existence of synthesizing enzyme(s);
(5) Existence of precursor molecules of the transmitter;
(6) Existence of a release mechanism and site of action for the transmitter;
(7) Identical actions among endogenous and exogenous transmitter;
(8) Pharmacological identity with other chemical substances.

### 5.1. Existence of Precursor Molecules and Synthesizing and Inactivating Enzyme(s)

As highlighted in Section 3, precursor molecules and synthesizing enzymes are involved in the production of β-alanine [11,20,24,26,27]. One pathway for β-alanine synthesis involves the conversion of precursor molecules 3-oxopropanoate (malonic semialdehyde) and L-alanine into pyruvic acid and β-alanine [11,20,24]. This process uses the enzyme β-alanine-pyruvate transaminase (β-alanine-α-alanine transaminase) [11,20,24]. Another pathway for β-alanine formation involves the precursor uracil [11,20,26,27]. Uracil is first converted into dihydrouracil, then into 3-ureidopropionate, and finally into β-alanine [11,20,26,27]. This reaction involves the enzymes dihydrouracil dehydrogenase (dihydropyrimidine dehydrogenase), dihydropyrimidinase (dihydropyrimidine amidohydrolase), and 3-ureidopropionase (β-alanine synthase) [11,20,26,27]. β-Alanine has precursor molecules and synthesizing enzymes [11,20,24,26,27].

Once β-alanine has completed its function, inactivating enzymes catabolize it [44,45,46,49]. β-Alanine can be converted into MSA by two enzymes: 4-aminobutyrate-2-oxoglutarate transaminase (also called GABA-T or β-alanine-2-oxoglutarate transaminase) and alanine-glyoxylate transaminase (also known as AGXT2 or β-alanine-pyruvate transaminase) [44,45,46]. Additionally, β-alanine can be transformed into AMM by pyruvate carboxylase [49]. Evidence supports the criterion that β-alanine interacts with enzymes that inactivate it [44,45,46,49].

### 5.2. Existence and Storage of the Transmitter in Neural Tissues

β-Alanine is transported into neural tissues [12,13,14,15,16,17]. This occurs through active or carrier-mediated transport across the BBB using a “beta-shuttle” [12,13,14,15,16,17]. β-Alanine is stored in multiple regions of the CNS [98]. Martin del Rio et al. determined the concentration of β-alanine in the rat CNS (*n* = 2–6). The highest concentration of β-alanine was found in the midbrain, near the cerebral cortex and medulla oblongata. β-Alanine was also detected in the olfactory bulb and tract, pons, hippocampus, spinal cord, and cerebellum [98]. There is no explicit measure of brain concentration in humans. While rodent studies have yielded interesting results, there is no brain concentration to enable rigorous analysis of pharmacological effects in humans. Furthermore, β-alanine is a precursor to carnosine, and although it can cross the BBB, most carnosine is synthesized in the brain [25]. This illustrates that it must be stored in the brain for synthesis to occur [20,28,29,37,44,99]. β-Alanine is also suggested to be stored in vesicular GABA transporter (VGAT) [100]. It is evident that β-alanine is present and stored in the brain [12,13,14,15,16,17,18,20,25,28,29,37,44,98,99,100].

### 5.3. Existence of a Release Mechanism and Site of Action for the Transmitter

**Figure 3:**
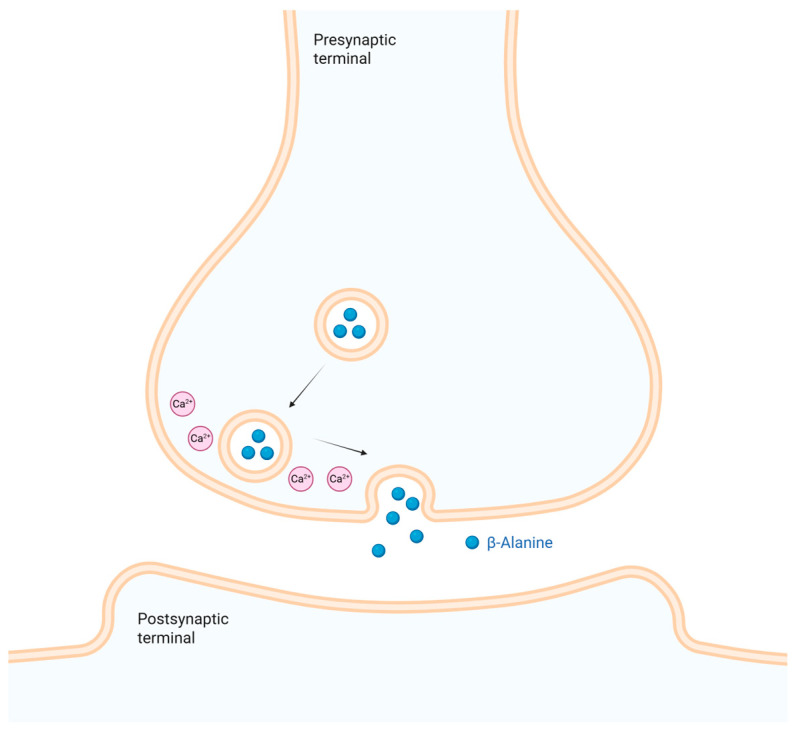
β-alanine is released from presynaptic neurons via a Ca^2+^-dependent mechanism. β-alanine then enters the synaptic cleft and can bind to the postsynaptic terminal, possibly contributing to synaptic dysfunction observed in AD [101,102]. Created in BioRender. Wozniczka, C. (2026) https://BioRender.com/32k8fo0.

The release mechanism of β-alanine in neurons has been widely studied [101,102]. Potassium (K^+^)-induced depolarization causes β-alanine to be released from presynaptic neurons through a calcium (Ca^2+^)-dependent process (Figure 3). It then diffuses across the synaptic cleft to the postsynaptic neuron in rabbit superior colliculus (SC) slices [101,102]. Juge et al. suggest that VGAT exocytoses β-alanine into the synaptic cleft, demonstrating another possible mechanism for β-alanine release [100]. This demonstrates that β-alanine possesses many possible release mechanisms [100,101,102].

Additionally, β-alanine acts at multiple sites [11]. These include the glycine co-agonist site on the NMDA complex (strychnine-insensitive), the glycine receptor site (strychnine-sensitive), the GABA-A receptor, the GABA-C receptor, and the GAT proteins. β-alanine targets many specific sites [11].

### 5.4. Identical Actions Among Endogenous and Exogenous Transmitter

Whether β-alanine is produced endogenously in the body or introduced externally, it affects the CNS in the same way [11,66,101,102,103]. Endogenous β-alanine in rabbit SC slices is released by the presynaptic neuron into the synaptic cleft, where it binds to one of its five receptor sites, resulting in inhibitory neurotransmission [11,101,102]. When exogenous β-alanine is administered to cat spinal motoneurons, inhibitory postsynaptic potentials are observed [66,103]. This demonstrates that β-alanine has the same inhibitory effect in neurons whether it is endogenous or exogenous [11,66,101,102,103].

### 5.5. Pharmacological Identity with Other Chemical Substances

β-Alanine functions similarly to other neurotransmitters, such as GABA and glycine [60,62,63,69,73,78,79,80,100,104,105,106]. β-Alanine mimics GABA by binding to the GABA site on the GABA-A receptor, and it can also mimic glycine by blocking the NMDA receptor, increasing inhibitory neurotransmission and decreasing neurotransmission, respectively. β-Alanine shares a pharmacological identity with other chemicals, particularly GABA and glycine [60,62,63,69,73,78,79,80,100,104,105,106].

### 5.6. β-Alanine Functions as a Neurotransmitter

β-Alanine meets the criteria to function as a neurotransmitter. Other authors have likewise concluded that β-alanine is a neurotransmitter, including Tiedje et al., Sandberg and Jacobson, Gemelli et al., Toggenburger et al., Komura et al., and DeFeudis and del Rio [11,101,107,108,109]. Recognizing β-alanine as a neurotransmitter facilitates its evaluation and consideration as a key molecular player in the pathogenesis and possible treatment of cognitive disorders such as AD, both as a supplement and as a platform for drug design.

## 6. β-Alanine Supplementation for Exercise Capacity and Cognitive Function

### 6.1. β-Alanine Supplementation in Humans

Numerous studies suggest that β-alanine supplementation may enhance cognitive function [15,99,110,111]. Generally, supplementing with β-alanine is linked to increased tolerance to post-traumatic stress disorder, mild traumatic brain injury, and heat stress [99]. Ostfeld et al. administered β-alanine for 10 weeks to older adults, resulting in improvements in cognitive abilities and a decrease in depression scores [15]. Ostfeld and Hoffman examined the effects of β-alanine supplementation in soldiers over periods ranging from 2 weeks to 6 months [99]. The findings showed increased resilience against post-traumatic stress disorder and mild traumatic brain injury [99]. Hoffman and colleagues administered β-alanine to individuals aged 60–80 years for 10 weeks, observing changes in brain inflammatory markers, such as brain-derived neurotrophic factor (BDNF) [110]. Imaging differences also appeared in the fractional anisotropy scores in the right hippocampus and left amygdala of the β-alanine group [110]. A systematic review by Cossu da Silveira analyzed articles published between 2014 and 2024 on the cognitive benefits of β-alanine supplementation in healthy people [111]. Several studies found no significant effects of β-alanine on cognitive function. However, other research suggested that β-alanine supplementation can improve cognitive abilities. These authors conclude that the existing evidence from 2014 to 2024 on the cognitive benefits of β-alanine in healthy individuals is limited [111]. There is evidence suggesting that β-alanine may enhance cognitive functions; however, further research is needed to confirm these findings [15,99,110,111].

### 6.2. β-Alanine and Carnosine Concentration Changes with Age

Sarcopenia is the loss of muscle mass that occurs with age, reducing the ability to engage in physical activity [112]. With age, muscle carnosine levels decline in both rats and humans [112]. When β-alanine is supplemented in elderly individuals, carnosine levels increase, which helps improve neuromuscular fatigue and exercise capacity [113]. For example, Stout et al. supplemented elderly individuals with β-alanine for 90 days [112]. It was found that this intervention could enhance exercise capacity by delaying the onset of neuromuscular fatigue [112]. Additionally, del Favero et al. administered β-alanine to elderly individuals for 12 weeks and observed increased carnosine levels and improved exercise capacity [114]. Other studies have shown that in healthy middle-aged people, β-alanine supplementation increases muscle carnosine levels [41,115]. Further research involving athletes and trained rowers who took β-alanine for 7 weeks demonstrated improved performance and higher carnosine levels [116]. Carnosine and β-alanine levels may vary with age; therefore, supplementing with β-alanine can boost carnosine in both young and older adults [41,112,113,114,115,116].

### 6.3. Consequences of Deficiency of β-Alanine and Carnosine in the CNS

β-Alanine levels in the CNS vary with age and health status [117]. When β-alanine levels in the CNS are low, the risk of neurodegenerative diseases increases. Hata et al. conducted a study among elderly Japanese residents of Hisayama, examining serum β-alanine levels in relation to dementia and AD. The results showed that higher serum β-alanine levels were significantly linked to lower risks of dementia and AD [117].

A deficiency of β-alanine in the CNS not only increases the risk of neurodegenerative diseases but also causes other effects [17,118,119,120,121]. For example, supplementing with β-alanine is linked to higher brain carnosine levels [17]. Lower levels of carnosine compromise neuroprotection, rendering the brain more susceptible to oxidative stress [17]. Boldyrev et al. suggest that carnosine could offer therapeutic benefits by helping to reduce oxidative stress in Alzheimer’s and Parkinson’s diseases [118]. Other studies show that carnosine has positive effects on diseases related to the CNS [119,120,121]. For instance, twelve weeks of carnosine supplementation in patients with Gulf War Illness (GWI) improved cognitive function [119]. Carnosine was also identified as a potential treatment for executive dysfunction in individuals with schizophrenia [120]. Fedoroca et al. propose that carnosine enhances baseline therapy for patients with Discirculatory Encephalopathy [121]. The deficiency of carnosine in the CNS is linked to neurodegenerative diseases; therefore, carnosine presents potential as a therapeutic option, similar to β-alanine [17,118,119,120,121].

The levels of β-alanine and carnosine may vary with age, and taking β-alanine supplements can raise carnosine concentrations in both young and elderly people [41,112,113,114,115]. Therefore, a deficiency in β-alanine and carnosine is associated with a higher risk of neurodegenerative disorders, indicating that these molecules or analogues thereof may be a potential therapeutic agent [17,117,118,119,120,121].

## 7. β-Alanine Role in Pathogenesis and Possible Treatment of AD

These sections outline various mechanisms, arranged from the most to the least probable as potential target sites for β-alanine. This section summarizes data from various studies, each with its own strengths and limitations. The evidence supporting β-alanine as a promising target remains strong, though additional research is necessary.

### 7.1. Neuronal Hyperexcitability and β-Alanine

Excessive activation of neuronal networks may result in excitotoxicity as a contributing pathogenic factor in AD [122]. Patients with AD are more prone to developing epilepsy and seizures as a manifestation of excess neuronal excitability [122]. Notably, the risk of clinical seizures is elevated by 6–10 times in individuals with sporadic AD [123]. Additionally, neuronal hyperexcitability is present in patients with MCIs, who are at a greater risk for the development of AD [122]. Two hypotheses have been proposed for the cause of neuronal hyperexcitability in epilepsy and AD [124,125,126,127,128,129,130]. The first hypothesis, known as the mossy fibre sprouting hypothesis, posits an increase in excitatory activity between neurons in hyperactive conditions [124,125,126]. This increase in excitatory activity in the brain is due to an increase in glutamate binding to NMDA receptors [131,132,133,134,135]. The second hypothesis, known as the dormant basket cell hypothesis, posits that a decrease in inhibitory activity between neurons results from dysfunction of GABAergic interneurons [128,129,130]. It is postulated that hyperexcitability results from increased excitatory glutamatergic synaptic transmission and decreased inhibitory GABAergic synaptic transmission [124,125,126,127,128,129,130,131,132,133,134,135,136,137,138,139,140,141]. These findings demonstrate that neuronal hyperexcitability participates in the pathology of AD and arises from an imbalance between excitatory and inhibitory processes.

Neuronal hyperexcitability is an evolving area of AD research, characterized by a GABA deficiency and an excess of glutamate. β-Alanine can therapeutically address neuronal hyperexcitability by targeting both anti-excitatory and pro-inhibitory activities within the brain [11]. β-Alanine binds as an agonist to the GABA site of the GABA-A receptor and as an antagonist to the NMDA glutamate receptor [64,106,142,143]. Thus, β-alanine has the potential of ‘hitting’ both of these receptors to increase neuronal inhibition by binding to GABA-A receptors and to decrease neuronal excitability by binding to NMDA receptors.

### 7.2. Amyloid-β (Aβ) and Tau Protein Aggregation and β-Alanine

Proteopathic aggregation of Aβ and tau is the time-honoured approach to AD [144]. β-Alanine can interact with Aβ and tau independently to prevent the aggregation of Aβ and tau [144]. A study by Preston et al. examined the effects of different pharmaceutical agents on the neurotoxic Aβ peptide fragment (Aβ25-35) in rat brain vascular endothelial cells [145]. Carnosine and β-alanine were among the agents that demonstrated cell rescue by inhibiting neurotoxicity caused by misfolded proteins [145]. Therefore, β-alanine is suggested as a potential inhibitor of the aggregation of both Aβ and tau proteins.

The mechanism by which β-alanine might inhibit Aβ/tau misfolding and oligomerization is speculative. Aβ plaques and tau tangles share a common AXBBXB hexapeptide motif where A represents any acidic residue, B is any basic residue, and X is any residue that is neither acidic nor basic; the common amino acid sequences for Aβ and tau are EVHHQK and DDKKAK, respectively [144]. Each amino acid sequence is important to target when addressing Aβ and tau protein aggregation for AD. The identification of a common AXBBXB hexapeptide motif within Aβ and tau enables a single β-amino acid, such as β-alanine, to target this motif [144]. Computational predictions suggest that β-alanine can bind to this motif within both Aβ and tau, likely due to its zwitterionic properties (at physiological pH) and has a two-carbon bridge between the ammonium and carboxylate termini. The positive and negative motifs of the misfolding peptides can interact with the positive ammonium group and the negative carboxylate group on β-alanine. β-alanine may help in reducing protein aggregation associated with AD.

### 7.3. Neuroinflammation and β-Alanine

Similar to protein aggregation, neuroinflammation plays a significant role in the pathogenesis of AD even before the symptoms of AD emerge [146]. Neuroinflammation is defined as an inflammatory reaction in the CNS that includes microglial activation and the release of pro-inflammatory cytokines [146,147,148,149]. Microglia play a crucial role in the innate immune response by protecting neurons subjected to damage while maintaining homeostasis [146,147,148,149,150,151]. Microglia are the macrophages of the CNS. Activated microglia accumulate near neurons and are the main contributors to neuroinflammation in AD [146,147,148,149,150,151]. Microglia become activated by pathogens and the misfolding of proteins such as Aβ and tau [149,150]. Microglia assume an activated phenotype to protect the CNS from pathogens represented by pathogen-associated molecular patterns (PAMPs), and damage-associated molecular patterns (DAMPs) [146,152,153,154,155,156,157,158]. These activated microglia stay in this phenotype and damage neurons by causing the release of pro-inflammatory cytokines such as tumor necrosis factor-α (TNF-α), and interleukin-1β (IL-1β) [146,149,152,153,154,155,156,157,158,159]. Activated microglia and the pro-inflammatory cytokines released by these microglia are the cellular and molecular basis of neuroinflammation in AD brains [146,147,148,149,150,151]. It is important to target these activated microglia and pro-inflammatory cytokines when finding a therapeutic approach for AD.

Activated microglia can release proinflammatory cytokines that contribute to neuroinflammation [146,149,152,153,154,155,156,157,158,159]. Several studies have evaluated the use of α-amino acids to inhibit pro-inflammatory cytokines and reduce neuroinflammation [160,161,162]. For example, Feijó et al. studied the effects of branched-chain amino acid (BCAA) supplementation on obese rats [160]. Obese rats exhibit increased IL-1β immunoreactivity in two brain regions: the cerebral cortex and the hippocampus. When these rats were supplemented with BCAAs, IL-1β immunoreactivity in the hippocampus decreased [160]. In alternative studies, α-amino acids have been shown to have regenerative properties on microglia/macrophages, altering neuroinflammation and remyelination [161,162]. There are also numerous studies suggesting that β-amino acids reduce the release of pro-inflammatory cytokines [163,164,165,166]. For example, Luo et al. analyzed multi-omics datasets to uncover the role of β-alanine metabolism in cellular inflammation in lipopolysaccharide (LPS)-stimulated RAW264.7 cells [163]. Chen et al. examined how adding 300, 600, or 1200 mg/kg of β-alanine to the diet of weaned piglets influences their immune response [164]. The results showed increased levels of immunoglobulin G (IgG), immunoglobulin M (IgM), and complement 3 (C3) in the piglets’ serum. They also observed a decrease in serum interleukin-6 (IL-6) levels in piglets in the 1200 mg/kg group [164]. Jin et al. investigated IL-6 levels in male college students who took 500 mg of β-alanine (two 250 mg capsules) daily for 4 weeks after long-distance exercise [165]. The findings indicated that IL-6 levels decreased following long-distance exercise [165]. Finally, Turcu et al. conducted a study measuring IL-6 levels in elite male basketball players after 8 weeks of β-alanine supplementation [166]. These players experienced a significant decrease in IL-6 levels during this period [166]. Based on predictive and mechanistic studies, amino acids such as β-alanine are expected to modulate pro-inflammatory cytokine release, which may inhibit neuroinflammation [163,164,165,166].

### 7.4. Reactive Oxygen Species (ROS) and β-Alanine

ROS are another factor contributing to the pathogenesis of AD [167,168,169,170,171,172,173,174,175,176,177,178,179,180,181,182,183]. ROS are molecules made of one or more oxygen atoms and/or free radicals; examples include superoxide anion (O^2−^), hydroxyl radical (HO•), and hydrogen peroxide (H_2_O_2_) [167]. ROS are produced as a byproduct of many endogenous processes, such as mitochondrial oxidative metabolism, and exogenous substances, including xenobiotic molecules. Oxidative stress happens when ROS are generated too rapidly for the cell to eliminate them [167]. This stress causes cellular damage, including damage to neurons, which can help lead to AD [167,168,169,171,172,173,174]. There is also evidence suggesting that ROS is linked to the formation of Aβ and tau aggregates and neuroinflammation [175,176,177,178,179,180,181,182,183]. ROS causes oxidative stress, which can contribute to the development and worsening of AD [167,168,169,170,171,172,173,174,175,176,177,178,179,180,181,182,183].

ROS play a crucial role in AD, and one potential approach to target them is through β-alanine and carnosine. β-Alanine and carnosine can act as antioxidants, neutralizing ROS [55,172,184,185,186,187,188,189,190,191,192,193,194,195,196]. Billacura et al. demonstrated that supplementing with β-alanine protects against metabolic stress in insulin-1 (INS-1) pancreatic β-cells and C2C12 skeletal muscle myotubes by reducing ROS [194]. Li et al. supplemented Yili horses participating in speed racing with β-alanine [193]. The total antioxidant capacity was found to increase both before and after racing in these horses. Additionally, an oxidation product known as malondialdehyde (MDA) decreased after racing in supplemented horses [193]. Smith-Ryan et al. analyzed blood samples from twenty-five men when supplemented with β-alanine [196]. The total antioxidant capacity was measured, leading to the conclusion that β-alanine has a minor antioxidant effect [196]. Belviranli et al. reached a similar conclusion, indicating that β-alanine and creatine supplementation demonstrated minor antioxidant effects during repeated Wingate tests in sedentary males [191]. Smith et al. studied the effects of β-alanine supplementation on oxidative stress [195]. Twenty-four women received either β-alanine or a placebo and completed a 40 min treadmill run to induce oxidative stress. The results showed significantly improved exertion levels after the treadmill exercise [195]. Additionally, a systematic review and meta-analysis by de França et al. examined studies on the effects of β-alanine and carnosine supplementation on exercise-induced oxidative stress [192]. These studies suggest that β-alanine and carnosine supplementation increase total antioxidant capacity and reduce pro-oxidant markers [192]. Furthermore, Gasmi et al., Jukić et al., Prokopieva et al., Reddy et al., Cao et al., Tanaka et al., Caruso et al., and Pekcetin et al. have suggested that carnosine has antioxidant properties [55,184,185,186,187,188,189,190]. β-Alanine and carnosine can serve as antioxidants to neutralize ROS, which may contribute to the development of AD [55,172,180,184,185,186,187,188,190,191,192,193,194,195,196].

### 7.5. Metal Dyshomeostasis and β-Alanine

Metal dyshomeostasis within cells can either be directly cytotoxic or may also stimulate the production of ROS by catalyzing the reaction of O_2_ into ROS such as O^2−^, HO•, and H_2_O_2_ [197]. These mechanisms occur in AD; multiple studies indicate that metal dyshomeostasis is involved in the pathogenesis of AD. For example, metal dyshomeostasis contributes to Aβ aggregation by activating γ-secretases and inhibiting α-secretases [198]. It also promotes tau hyperphosphorylation through the activation of protein kinases like cyclin-dependent protein kinase-5 (CDK5). Furthermore, metal imbalance can affect organelles such as the endoplasmic reticulum (ER) and mitochondria, further facilitating protein aggregation and impairing synaptic functions [198].

There is also metal dyshomeostasis of many metal ions including copper (Cu^2+^), zinc (Zn^2+^) and iron (Fe^2+^/Fe^3+^) ions involved in AD pathogenesis [198,199,200,201,202,203,204,205,206,207,208,209,210,211,212,213,214,215,216,217,218]. Some studies suggest Cu^2+^ deficiency, while others indicate an excess of these ions plays a role in AD progression [199,200,201,202,203]. For example, Xu et al. found that brain regions impaired by AD had Cu^2+^ deficiency [203]. Conversely, Squriti et al. detected higher serum Cu^2+^ levels in AD subjects compared to controls [202]. Nonetheless, extracellular Cu^2+^ binds to Aβ, promoting its aggregation into toxic fibrils and plaques, which induce ROS generation and oxidative stress [201]. Intracellular Cu^2+^ also affects tau protein conformation and kinases involved in neurofibrillary tangle formation [201]. Similar to Cu^2+^ ions, AD has been associated with both increased and decreased intracellular Zn^2+^ levels [204,205,206,207,208,209,210,211,212]. Religa et al. demonstrated a twofold increase in Zn^2+^ ions in the cortex of AD patients [212]. They also noted higher Zn^2+^ levels in Aβ-rich regions [212]. Conversely, Rivers-Auty et al. suggest that zinc deficiency contributes to cognitive decline via NLR family pyrin domain containing 3 (NLRP3)-driven inflammation in animal models of AD [204]. Brewer et al. found that serum zinc levels in AD patients are about 14% lower than in controls [210]. Yet, increased Zn^2+^ levels are known to bind to Aβ, facilitating its aggregation [207,208,209]. Additionally, an imbalance of Fe^2+^/Fe^3+^ ions is seen in AD [213,214,215,216,217,218]. Ayton et al. reported elevated Fe^2+^/Fe^3+^ in the inferior temporal cortex of AD and dementia patients [213]. Bartzokis et al. also showed increased Fe^2+^/Fe^3+^ in the basal ganglia, particularly the caudate and putamen, using magnetic resonance imaging [214]. Therefore, metal dyshomeostasis is a critical factor in the progression of AD [198,199,200,201,202,203,204,205,206,207,208,209,210,211,212,213,214,215,216,217,218].

Metal dyshomeostasis plays a role in AD development [167,168,169,170]. Therefore, efforts have focused on regulating the levels of Cu^2+^, Zn^2+^, and Fe^2+^/Fe^3+^ ions within cells to prevent the catalysis of O_2_ into ROS. Research has concentrated on developing chelators, molecules with ligands that bind metal ions, specifically targeting Cu^2+^, Zn^2+^, and Fe^2+^/Fe^3+^ ions as potential agents to inhibit ROS-mediated cytotoxicity [219]. Many chelators are amino acid derivatives used in medicine, water softening, and soil cleanup [220,221]. Past research also explored the use of metal chelators as a therapy for AD [222]. Specifically, Pulido et al. used β-alanine as a Cu^2+^ chelator in *Caenorhabditis elegans* (*C. elegans*) brain to test whether ROS production was reduced [223]. Their results found reduced ROS. Additionally, Cu^2+^ chelation with β-alanine enhanced the lifespan of *C. elegans* that had Aβ in their brain, suggesting that Cu^2+^ chelation may also alter Aβ protein misfolding [223]. It is predicted that the negatively charged carboxylate ion in the β-alanine zwitterion can act as a ligand to bind to the positively charged Cu^2+^, Zn^2+^, and/or Fe^2+^ ions, facilitating chelation and preventing the catalytic conversion of O2 into ROS, thereby reducing neuronal damage. Studies also indicate that carnosine can chelate metal ions such as Cu^2+^, Zn^2+^, and Fe^2+^/Fe^3+^ [224,225,226,227,228]. This capability might be due to the presence of L-histidine in carnosine, which contains an imidazole ring likely involved in metal binding, along with β-alanine [28,29]. Overall, β-alanine and carnosine are expected to be useful in managing Cu^2+^, Zn^2+^, and Fe^2+^/Fe^3+^ chelation to address metal dyshomeostasis in AD.

β-alanine is hypothesized to target multiple known factors in the progression and development of AD, specifically neuronal hyperexcitability, Aβ and tau aggregation, neuroinflammation, ROS, and metal dyshomeostasis.

### 7.6. Taurine’s Connection to the Pathogenesis of AD and β-Alanine

Taurine (2-aminoethanesulfonic acid) is a common amino acid in the human body, especially in the brain, retina, and muscle tissue; it is a structural analogue of β-alanine [229,230,231]. Taurine can be obtained from animal products [231]. Similar to β-alanine, taurine uses Tau-T and PAT1 transporters to move from the stomach to the liver and then into the bloodstream [231]. Taurine can also be biosynthesized in the liver by converting methionine to cysteine-sulfonic acid, and eventually to hypotaurine and taurine [232]. It is also produced in the brain, particularly in the hippocampus and cerebellum, where cysteine is converted by sulfonic acid decarboxylase (taurine-synthase) into taurine [232]. Taurine made outside the brain can cross the BBB via high-affinity Na^+^- and Cl^−^-dependent transporters, such as TauT and GAT-2 [233]. Taurine plays many roles in the CNS, including cryoprotection, bile acid conjugation, calcium balance, osmoregulation, and membrane stabilization [229,230,234]. It also meets most of the criteria for being considered a neurotransmitter [229,234]. The body excretes most of the excess taurine unchanged through glomerular filtration in the kidney or by conjugation with bile acids in the liver [231]. Taurine is a non-essential amino acid that can be obtained through diet or produced in the body and has many functions in the CNS [229,230,231,232,233,234].

Taurine plays a crucial role in the human body, similar to β-alanine. Both molecules share similar chemical structures, with the main difference being the substitution of the anionic sulfonic acid moiety in taurine with a bioisosterically equivalent anionic carboxylic acid moiety in β-alanine. Significantly, sulfonic acid is a highly polar functional group. Drugs with lower polarity (and higher lipophilicity) are more likely to cross the BBB [235]. Replacing sulfonic acid with a carbonyl group reduces polarity, which could be advantageous in drug development for AD.

**Figure 4:**
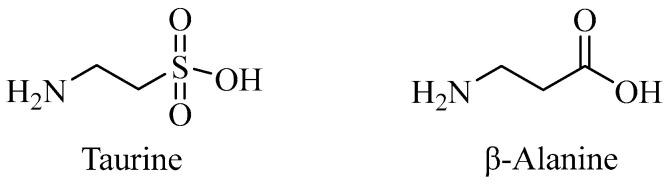
The structure of β-alanine compared to taurine. Taurine and β-alanine are structurally similar to one another. Taurine has a sulfur group, whereas β-alanine has a carbonyl group. Evidence more strongly supports taurine’s relevance in AD compared to β-alanine.

Taurine and β-alanine (Figure 4) are chemically similar, indicating their analogous potential as putative therapeutic platforms for AD. For instance, Kim et al. administered taurine orally to adult APP/PS1 mice for six weeks, resulting in improved cognitive deficits [236]. Similarly, Oh et al. gave FAx5 AD transgenic mice taurine daily, resulting in increased brain uptake of glutamate receptor type 5 (MGLU5) [237]. Taurine also targets the same mechanistic pathways involved in AD pathogenesis as β-alanine. Chen et al. (b) propose that taurine boosts cognitive function through various mechanisms, such as activating GABA-A receptors, reducing neuroinflammation, increasing Nrf2 expression, enhancing antioxidant capacity, and lowering oxidative stress [238]. Overall, taurine may influence AD pathogenesis by acting at shared receptor sites similar to those of β-alanine. Thus, many studies on the potential therapeutic utility of taurine in AD support the possible role of β-alanine as a structural analogous therapeutic for AD.

#### 7.6.1. Neuronal Hyperexcitability and Taurine

Experimental studies demonstrate that taurine modulates neuronal hyperexcitability [239,240,241,242,243,244,245,246,247]. Lee et al. demonstrated that taurine has a protective effect on cultured motor neurons and prevents neurotoxic injury [239]. L’Amoreaux et al. found that mice fed taurine were less likely to experience picrotoxin-induced seizures [240]. These authors suggest that taurine alters the GABA-A receptor and decreases the amount of picrotoxin that can bind to it [240]. Jia et al. discovered that, at physiological concentrations of taurine, ventrobasal (VB) thalamus neurons were inhibited due to the activation of GABA-A receptors [241]. Ochoa-de la Paz et al. state that taurine is an agonist of the GABA-A receptor, and the affinity for this molecule depends on the subunits that compose the GABA-A receptor [242]. Bureau et al. demonstrated that taurine binds to the GABA-A receptor at the GABA site in mammalian brain regions during in vitro experiments [243]. Chan et al. conducted electrophysiological and receptor binding experiments on taurine’s interaction with NMDA receptors [244]. They found that taurine decreases the affinity of glycine for the NMDA receptor [244]. Paula-Lima et al. propose that neurons exposed to high extracellular glutamate concentrations, which cause excitotoxicity, can be rescued by taurine [245]. Chan et al. (b) performed electrophysiological and receptor binding studies on layer 5 of the prelimbic cortex in rat brain slices to examine how taurine interacts with NMDA receptors [246]. There was a change in NMDA receptor function when taurine was applied, and taurine reduced the affinity of glycine for the NMDA receptor [246]. Suárez et al. showed that taurine inhibited the effect of 7-chlorokynurenic acid on NMDA-induced potentials [247]. This demonstrates that taurine can block the glycine site on the NMDA receptor with high affinity [247]. Overall, taurine can reduce neuronal hyperexcitability mainly by altering GABA-A and NMDA receptors, similar to β-alanine [239,240,241,242,243,244,245,246,247].

#### 7.6.2. Aβ and Tau Protein Aggregation and Taurine

Taurine impacts the two main pathological processes in AD: Aβ accumulation and tau aggregation [245,248,249,250,251,252,253]. Paula-Lima et al. show that micromolar doses of taurine prevent Aβ buildup in cultured rat hippocampal and cortical neurons [245]. Louzada et al. found that taurine helps reduce Aβ neurotoxicity in cultured chick retinal neurons [248]. Lee et al. (b) observed that 10–100 μM of taurine decreased in vitro Aβ42 fibril formation using transmission electron microscopy [249]. The fibrils appeared looser at higher taurine levels. Computer simulations by the same researchers indicated that taurine binds to Aβ peptide fragments and can break apart Aβ dimers. HT22 cells treated with taurine showed less Aβ cell toxicity. When given orally to 5XFAW mice for 4 weeks at 1000 mg/kg, taurine reduced Aβ accumulation and tau hyperphosphorylation in the dorsal subiculum [249]. Jang et al. demonstrated that taurine administration improves cognitive function by binding to oligomeric Aβ in AD mouse models [250]. Abdulkadir et al. showed that taurine decreases Aβ aggregation in female Wistar rats after 8 weeks of oral supplementation [251]. Jahanshahi et al. dosed adult male Wistar rats with different taurine amounts, resulting in lowered phosphorylated tau protein levels in the cerebellum and prefrontal cortex at the highest dose (100 mg/kg/day) [252]. Asadi et al. injected male Wistar rats daily with taurine (25, 50, 100 mg/kg) for 2 weeks; the 50 mg/kg group showed a significant reduction in hippocampal phosphorylated tau [253]. Taurine, like β-alanine, affects the two well-established pathogenic processes in AD: Aβ and tau aggregation [245,248,249,250,251,252,253].

#### 7.6.3. Neuroinflammation and Taurine

Taurine can inhibit neuroinflammation, a known contributor to AD pathogenesis [249,254,255,256,257,258,259,260,261]. Ahmed et al. studied the brains of senescence-accelerated mouse prone 8 (SAMP8) mice to investigate taurine’s effect on activated microglia [254]. Their immunohistochemical and Western blot analyses showed that taurine reduced the number of activated microglia in the hippocampus and cortex. Additionally, taurine elevated triggering receptor expressed on myeloid cells 2 (TREM2) expression in these brain regions, which can decrease microglia overactivation [254]. Lee et al. (b) showed that taurine administration reduced microglial injuries and illustrated a decline in Iba-1 immunoreactivity [249]. Zhao et al. exposed adult male Sprague-Dawley rats to collagenase-induced intracerebral hemorrhage (ICH) injury and then supplemented them with varying concentrations of taurine [255]. This led to a reduction in inflammatory damage, marked by decreased glial cell activation, lower inflammatory markers, and reduced neutrophil infiltration [255]. Liu et al. (b) supplemented taurine to LPS-treated mice [256]. These mice exhibited reduced microglia activation in the hippocampus and a decline in IL-6, TNF-α, inducible nitric oxide synthase, and cyclooxygenase-2 [256]. Silva et al. studied LPS-induced neuroinflammation in the cerebellum of rats when taurine was administered orally [257]. Taurine was found to have antiapoptotic properties that can protect the rat’s cerebellum from neuronal death and neuroinflammation [257]. Che et al. discovered that both nicotinamide adenine dinucleotide phosphate (NADPH) oxidase (NOX2) and the nuclear factor-kappa B (NF-κB) pathway, involved in M1 microglial inflammation, are inhibited in a Parkinson’s disease mouse model [258]. Su et al. examined inflammatory cytokine release and astrocyte activity in rats after traumatic brain injury (TBI) when given taurine intravenously [259]. They found taurine lowered growth-regulated oncogene/keratinocyte chemoattractant (GRO/KC) and IL-1β levels after 24 h and reduced 17 cytokines, including TNF-α, IL-1β, and IL-6, after one week [259]. Chupel et al. demonstrated that exercise and taurine in elderly women reduced neuroinflammation and preserved BBB integrity [260]. Vahdat et al. analyzed serum levels of inflammatory markers in 32 TBI patients supplemented with taurine [261]. Taurine decreased serum IL-6 levels in these patients [261]. Overall, taurine has the capacity to suppress neuroinflammation, which significantly contributes to AD development, similar to β-alanine [249,254,255,256,257,258,259,260,261].

#### 7.6.4. ROS and Taurine

ROS are involved in AD development, and taurine is known to lower ROS levels as an antioxidant [256,262,263,264,265,266,267,268,269]. Liu et al. (b) and Lee et al. (c) showed a decrease in ROS in LPS-stimulated BV-2 and HT22 cells when treated with taurine, respectively [256,262]. Jong et al. explored how taurine modulates oxidative stress [263]. These authors found that taurine affects mitochondrial protein synthesis, enhances electron transport, and prevents mitochondrial superoxide production [263]. Abud et al. gave taurine to twenty-four women over 16 weeks to measure plasma oxidative stress markers [264]. A reduction in the antioxidant enzyme superoxide dismutase (SOD) was observed [264]. Teles et al. administered taurine to *Seriola rivoliana* juveniles after LPS treatment and evaluated antioxidant enzyme activity [265]. Supplementing this fish with 2% taurine increased IL-β, TNF-α, and toll-like receptor-3 (TLR-3) expression after 24 h of LPS treatment. It also boosted antioxidant and lysozyme activity in the taurine group [265]. Maleki et al. treated type 2 diabetes patients with taurine for 8 weeks, three times daily [266]. Results showed improved oxidative stress, with higher SOD and catalase enzyme activities and lower MDA and TNF-α levels [266]. Moreover, Shimada et al., Surai et al., and Jong et al. (b) indicate that taurine has antioxidant effects [267,268,269]. Taurine, like β-alanine, has antioxidant activity that helps reduce ROS levels, possibly in AD [256,262,263,264,265,266,267,268,269].

#### 7.6.5. Metal Dyshomeostasis and Taurine

Metal dyshomeostasis is a key factor in the pathogenesis of AD, and taurine is known to help regulate this imbalance [270,271,272,273,274,275,276,277]. Zhang et al. (b) revealed that taurine might help control Se, Cu, and Fe levels in the liver and kidneys of arsenic-induced mice [270]. O’Brien, Farkas, and Nolan show that taurine can form complexes with Cu^2+^ ions [271]. However, these complexes are not as stable and strong as when β-alanine binds to Cu^2+^ ions. The authors also suggest that taurine could bind Zn^2+^ under physiological conditions [271]. Tekin et al. conducted a study with thirty male rats to examine the effect of taurine supplementation on rats expressing Aβ 1-42 over six weeks [272]. Results showed decreased plasma Cu^2+^ and Aβ 1-42, and reduced brain and kidney levels of low-density lipoprotein receptor-related protein-1 (LRP-1) in the treatment group [272]. Choi et al. studied eighty *Carassius auratus* to analyze cadmium levels in muscle, gills, and bone tissue [273]. Cadmium concentrations decreased in the groups receiving taurine after cadmium exposure [273]. Liu et al. (c) showed that taurine supplementation reduced the harmful effects of ferroptosis, increased glutathione levels, lowered MDA, decreased ROS, and reduced Fe^2+^ levels in a myoblast ferroptosis model [274]. Wenting et al. demonstrated taurine’s neuroprotective properties in rats with aluminum-related neurological disorders [275]. Król, Okulicz, and Kupsz examined taurine supplementation in rats fed either a regular or high-fat diet [276]. They found that trace element levels varied between the groups. Rats on a high-fat diet given taurine had lower renal and spleen Zn levels, while rats on a regular diet given taurine had lower renal Cu levels [276]. Oudit et al. showed that taurine could reduce iron-mediated oxidative stress in the myocardium of a mouse model [277]. Taurine is widely recognized for its role in helping to regulate this imbalance of metal levels, similar to β-alanine [270,271,272,273,274,275,276,277].

Taurine, a structural analogue of β-alanine, interacts with the same sites involved in AD pathogenesis, including neuronal hyperexcitability, Aβ and tau aggregation, neuroinflammation, ROS, and metal dyshomeostasis. These parallel bioactivities of the two principal β-amino acids in the human brain justify studying β-alanine as a neurotransmitter and a druggable molecular platform for the development of small-molecule therapeutics for AD.

### 7.7. Comparison of β-Alanine to Other Amino Acids and Multimodal Approaches in AD

This review indicates that β-alanine could serve as a drug target for modifying the development of AD. To highlight the differences in β-alanine compared to other molecules and amino acids, a simple comparison with amino acids like L-serine, L-tryptophan/5-HTP, and L-arginine is shown in Table 2 [278,279,280,281,282,283,284,285]. L-serine, L-tryptophan, and L-arginine differ substantially from β-alanine in their biological roles and in the extent to which they have been shown to help treat AD [278,279,280,281,282,283,284,285]. L-serine, which is converted to D-serine, binds to the NMDA co-agonist site and increases activity at NMDA receptors [278]. This increases excitatory neurotransmission in the CNS; thus, higher levels of L-serine/D-serine are associated with increased neuronal hyperexcitability, leading to neurodegenerative effects in AD animal models [278,279,280]. The opposite effect has been documented for β-alanine, where β-alanine can bind to the NMDA co-agonist site and block D-serine from binding [11]. Moreover, AD animal models supplemented with high L-tryptophan diets are shown to alter Aβ density [282]. There are no studies suggesting any alteration in Aβ density; it is only a speculation that β-alanine could indirectly alter Aβ protein aggregation [144]. Furthermore, L-arginine is involved in nitric oxide production, which may provide direct cognitive benefits in AD [283,284,285]. There is no direct link between β-alanine and altering NO levels. Still, there is direct evidence supporting the antioxidant effect of β-alanine and carnosine, which may provide cognitive benefits [55,172,184,185,186,187,188,189,190,191,192,193,194,195,196]. None of these amino acids, including β-alanine, are currently established as a treatment for AD. However, β-alanine differs structurally from the others and may target multiple receptor sites in AD.

It is also essential to clarify how β-alanine integrates into current multimodal treatments for AD. Table 3. illustrates this, including combinations like memantine with donepezil, and two multitherapeutic molecules, memoquin and ladostigil [6,7,286,287,288,289]. The existing interventions, such as symptomatic relief agents like memantine and donepezil, have been combined for AD treatment [286]. The clinical use of memantine and donepezil together has demonstrated short-term symptomatic benefits with acceptable safety and tolerability [6,7,286]. Nonetheless, both agents are acknowledged as not altering AD progression [6,7,286]. Two molecules demonstrating polypharmacology, memoquin and ladostigil, have been shown to influence AD pathogenesis in preclinical models [287,288,289]. Yet, memoquin has not progressed to a clinical trial, and ladostigil has failed in clinical trials to slow down the progression of AD [287,288,289]. The agents described above provide evidence that multimodal therapies have been developed for AD, but none have yet produced a successful treatment that can change the course of the disease. Currently, β-alanine is still far from having any clinical significance, but it is a molecule with a novel structure that targets multiple pathways in AD development. β-Alanine occupies a new and unique position among multimodal AD therapeutics, making it a potential drug target for AD pathogenesis [6,7,286,287,288,289].

### 7.8. Druggability of β-Alanine

β-Alanine, the simplest β-amino acid, is a small, structurally uncomplicated molecule suitable as a starting point for the synthesis of analogues. For example, Tan et al. demonstrate that this molecule can be modified at the N-, α-, and β-positions using synthetic routes that require no more than four steps. This shows that synthesizing β-alanine analogues is achievable and can be successfully performed to produce a range of structurally diverse compounds that cover the chemotype space defined by this unexploited neurotransmitter.

To be developed as putative agents for AD, these analogues must also cross the BBB to serve as potential drug candidates. They should cross the BBB through active transport, and this process needs to be evaluated individually for each analogue. Each analogue will also need to be tested in various in vitro assays targeting neuronal hyperexcitability, Aβ and tau aggregation, neuroinflammation, ROS, and metal dyshomeostasis to determine its activity. Continued research on β-alanine analogues with different substitutions is necessary to find a therapeutic approach for AD. As mentioned in this review, β-alanine is an understudied neurotransmitter; additional key studies will be crucial for the continued development of the target as a druggable neurotransmitter.

## 8. Limitations

Should β-alanine and its analogues be considered as drug targets for AD? The incorporation of polypharmacology for each mechanistic site into a single β-alanine is challenging. Optimizing the structure–activity relationship (SAR) for each mechanistic site is a complicated, time-consuming, and costly process. Additionally, β-alanine and its analogues must also cross the BBB. Each analogue designed, synthesized, and assessed will also need to be evaluated to determine if it can cross the BBB via active transport. It is clear that this molecular platform has many limitations that must be considered before undergoing the synthesis and testing of β-alanine analogues. All the data presented are from in vitro and in vivo studies, and there is limited current evidence on β-alanine’s ability to target AD pathogenesis within a human clinical setting. This would involve having a confirmed mechanism of action with associated proof of target engagement. Furthermore, as this research advances toward a clinical candidate, it would be crucial to consider other limitations. These include dose dependence, safety, BBB saturation, and translational feasibility.

### 8.1. Dose Dependence

β-Alanine is not an approved treatment for AD, and its dose–response relationship in this particular context has not been established. Current evidence indicates β-alanine can reliably raise carnosine levels, thereby improving neuromuscular fatigue at standard supplemental doses (2–6 g/day), with minimal side effects in healthy people [290]. Preliminary research also indicates possible cognitive advantages for healthy individuals, though evidence is lacking for those with diagnosed AD, and precise dose–response data are unavailable [15,99,110,111]. More rigorous research, like controlled clinical trials involving early AD or MCI groups with clear endpoints, is needed to determine whether specific dosing guidelines can be established.

### 8.2. Safety

Typically, β-alanine is safe for healthy adults at doses up to about 6.4 g per day, according to exercise science research, with tingling (paresthesia) as the primary side effect, depending on the dose [290,291]. However, these safety finding do not account for AD patients or long-term neurological use. There is no confirmed maximum dose for neuroprotective uses. Effects on brain-specific endpoints, such as long-term cognitive decline, amyloid burden, and tau pathology, have not been sufficiently studied to determine safe dose ranges.

### 8.3. BBB Saturation

In humans, β-alanine crosses the BBB through a saturable, low-capacity, and tightly regulated process mediated by active or carrier-mediated transporters [12,13,14,15,16,17,18]. This regulation prevents significant buildup in the brain, maintains β-alanine balance, and limits CNS effects even when blood levels are high [12,13,14,15,16,17,18]. The brain is shielded from large spikes even when blood levels rise, which is a typical sign of a saturable BBB transporter [12,13,14,15,16,17,18].

### 8.4. Translational Feasibility

β-alanine has shown potential as a neuroprotective agent in AD in preclinical studies. However, transitioning to human use poses challenges, including identifying the correct dosage, and limited direct evidence. The next step should be pilot human trials focusing on cognitive outcomes and measuring brain β-alanine levels.

## 9. Conclusions

Current evidence and/or speculation suggest that β-alanine could influence targets involved in AD pathogenesis. These targets include neuronal hyperexcitability, protein aggregation, neuroinflammation, ROS, and metal dyshomeostasis. However, the overall evidence is limited and mainly indirect. Most data derive from preclinical models, metabolomic studies, or inferences from related neuroprotective processes, rather than direct evidence in AD. There is considerable uncertainty regarding brain availability, target specificity, long-term safety, and whether these effects are disease-modifying or simply symptomatic. Future research should focus on studying the molecular mechanisms of β-alanine’s targets in the AD-related pathways discussed in this review, conducting biomarker studies in humans, and performing in vivo experiments to assess cognitive and pathological outcomes. Ultimately, early clinical trials are essential to evaluate translational potential and therapeutic feasibility.

## Figures and Tables

**Table 1:** Brief summary of current therapeutic approaches for AD [6,7,8,9,10]. There are small-molecule drugs, specifically NMDA receptor antagonists and cholinesterase inhibitors, that only provide symptomatic relief [6,7]. There are also monoclonal antibodies, including Lecanemab and Donanemab, that reduce Aβ plaque formation [8,9,10].

Therapeutic Name	Mechanism of Action of Therapeutic	Limitations of Therapeutic	References
Memantine	NMDA Receptor Antagonists	Only symptomatic relief	[6]
Donepezil, Galantamine, and Rivastigmine	Cholinesterase Inhibitors		[7]
Lecanemab	Binds to amyloid oligomers, protofibrils, and insoluble fibrils	Cerebral microhemorrhages and vasogenic cerebral edema	[8,9,10]
Donanemab	Binds to N-terminal pyroglutamate (N3pG) Aβ plaques, thereby reducing the buildup of this misfolded protein		

**Table 2:** A summary of other amino acids in terms of mechanisms and evidence related to AD pathogenesis.

Amino Acid	Mechanism	Evidence	References
L-Serine	A precursor for D-serine (NMDA co-agonist).	AD animal models demonstrate neurodegenerative effects in the presence of altered D-serine.	[278,279,280]
L-Tryptophan/5-HTP	A precursor to serotonin with receptors found on postsynaptic and presynaptic neurons.	AD animal models supplemented with high tryptophan reduced Aβ density.	[281,282]
L-Arginine	A precursor of nitric oxide that diffuses across the cell membrane.	AD animal models demonstrate neuroprotection when supplemented with arginine.	[283,284,285]

**Table 3:** A summary of multimodal approaches in AD that have achieved clinical success, those that did not reach clinical application, and those that failed to meet endpoints in trials.

Intervention	Evidence	Limitations	References
Memantine and Donepezil	The combination treatment showed a reduction in clinical worsening with reasonable safety and tolerability.	Benefits are small to modest, and this is only symptomatic relief and not disease modification.	[6,7,286]
Memoquin	In vitro and in vivo studies suggest this molecule can target multiple receptor sites involved in AD pathogenesis: ACh inhibition, anti-Aβ aggregation, and antioxidant properties.	No clinical data supporting in vitro and in vivo studies.	[287]
Ladostigil	Shown to target monoamine oxidaseA and B and cholinesterase inhibitory activities.	Primary endpoints not met in Phase II clinical trials.	[288,289]

## Data Availability

No new data were created or analyzed in this study. Data sharing is not applicable to this article.
